# Medullary Thyroid Carcinoma: Targeted Therapies and Future Directions

**DOI:** 10.1155/2009/183031

**Published:** 2009-12-24

**Authors:** Scott N. Pinchot, Muthusamy Kunnimalaiyaan, Rebecca S. Sippel, Herbert Chen

**Affiliations:** Endocrine Surgery Research Laboratory, Department of Surgery, University of Wisconsin, H4/722 Clinical Science Center, 600 Highland Avenue, Madison, WI 53792, USA

## Abstract

Medullary thyroid cancer (MTC) is a rare neuroendocrine neoplasm that accounts for approximately 5% of all thyroid malignancies. The natural history of MTC is characterized by early lymph node and distant metastases, making complete surgical cure often impossible. Conventional chemotherapy and external beam radiation have been largely ineffective in altering the natural history of MTC. Therefore, there is a great need to develop novel therapeutic strategies to affect symptom control and reduce tumor burden in patients with widely disseminated disease. Here, we review several pathways which have been shown to be vital in MTC tumorigenesis and focus on the pathways of interest for which targeted drug therapies are currently being developed.

## 1. Introduction

Medullary carcinomas of the thyroid are neuroendocrine neoplasms derived from the parafollicular cells, or C cells, of the thyroid and account for nearly 5–10% of thyroid malignancies [1]. In nearly all cases of medullary thyroid cancer (MTC), the C cells secrete calcitonin, a specific and highly sensitive biomarker whose measurement plays an important role in the diagnosis and postoperative followup of patients [2, 3]. Less common, MTC cells elaborate other polypeptide hormones, including vasoactive intestinal peptide (VIP), serotonin, somatostatin, and carcinoembryonic antigen (CEA), the latter of which has been shown to herald contralateral lymph node and distant metastases [4]. 

The majority of MTCs are sporadic, but up to 25% of MTC cases result from a germ-line activating mutation in the rearranged during transfection (*RET*) proto-oncogene [5, 6]. Hereditary MTCs occur in the setting of the multiple endocrine neoplasia (MEN) syndrome type 2 (2A or 2B) or as familial MTC (FMTC) without an associated MEN syndrome. In sporadic MTC, patients most commonly present in the fifth or sixth decade with a palpable cervical lymph node or a solitary thyroid nodule. Fine needle aspiration (FNA) biopsy of the mass and the presence of an elevated serum calcitonin are diagnostic of MTC. Unlike sporadic MTC, most patients with hereditary disease are identified by genetic testing of at-risk family members for the germline mutation of the *RET* gene. As such, hereditary MTC tends to present at an earlier age than sporadic disease and is typically multifocal and bilateral [1]. 

 The management of MTC relies heavily on surgical resection, consisting of total thyroidectomy and lymph node dissection; however, recurrent disease develops in approximately 50% of patients with MTC [7]. Similarly, biochemical documentation of persistent or recurrent MTC by serum calcitonin levels is often associated with unresectable recurrence in distant locations, including lung and liver [8]. Therefore, although MTC tends to be a slow-growing tumor primarily treated with surgical resection, it frequently metastasizes early in the disease course to the liver and regional lymph nodes, precluding patients from a curative resection. It is thus necessary to develop alternative therapeutic strategies to control tumor growth, possibly through manipulation of various cellular signaling pathways [9].

## 2. Molecular Pathogenesis and Cytogenetics

Although MTC is rare, there has been considerable interest in the molecular pathways that regulate MTC cellular growth, differentiation, survival, and hormone expression. We and others have previously shown that manipulation of these pathways may be a potential therapeutic strategy to control the growth and hormone production of NE tumors like MTC [9–12]. With the application of current molecular techniques, decades of research have begun to elucidate a genetic model that contributes to MTC tumorigenesis that includes three important processes: mutated proto-oncogenes which result in altered receptor protein production and concomitant accelerated tumor growth, alterations in signal transduction pathways which regulate the NE phenotype, and variations in tumor suppressor genes that facilitate unregulated cell growth [13]. Although a comprehensive review of all pathways which have been studied is beyond the scope of this paper, we aim to focus on new pathways of interest in MTC for which targeted drug therapies are currently in development (Table 1).

## 3. Receptor Proteins

### 3.1. The RET Receptor Tyrosine Kinase (RTK)

Activating germline mutations in familial MTC involve the *RET* proto-oncogene, which is mapped to chromosome 10q11.2 [1]. The *RET* gene encodes a 120-kDa transmembrane receptor tyrosine kinase (RTK) that functions as a target for the glial-derived neurotrophic factor (GDNF) family of growth factors [14]. Mutations in the *RET *proto-oncogenes have been implicated in nearly 95% of cases of hereditary MTC associated with MEN types 2A and 2B and FMTC. Interestingly, in hereditary MTC, recent data suggest that specific germline *RET* mutations are associated with age-specific penetrance of cancer development and lymph node metastases [2, 15]. The most common mutation in MEN 2A (codon 634) occurs in up to 80% of MEN 2 families and nearly half of affected children develop MTC by ages 5–10. The codon most frequently associated with MEN 2B (codon 918) confers a significantly higher risk of MTC, often beginning in the first 6 months of life. Patients assigned to this highest risk category clearly benefit from prophylactic thyroidectomy in the first year of life, if possible.

While MTC displays a slow-growth pattern and indolent disease course, frequent metastases to the liver and regional nodal basins plague patients with hereditary disease, precluding these patients from a curative resection. Clearly, there is a great need for novel therapeutic and palliative strategies to treat these patients with metastatic MTC. 

Several small molecule RTK inhibitors have been developed against RET and show promising results both in vitro and in vivo as emerging therapies for the treatment of MTC. These molecules include ZD6474 (Vendetanib), SU11248 (sunitinib), BAY 43-9006(sorafenib), CEP-751 and CEP-701, XL-880, XL-184, and RPI-1 [2, 16]. Much is known about vandetanib, a low molecular weight tyrosine kinase inhibitor that has demonstrated effective inhibition of RTK in vitro [17, 18]. Likewise, Carlomagno et al. [17] have shown that vandetanib blocks in vivo phosphorylation and signaling of the *RET*/MEN2B oncoprotein and prevents the growth of two human cancer cell lines that carry spontaneous *RET *rearrangements. Currently, promising data in patients with MTC have led to vandetanib being assigned orphan drug designation by the US Food and Drug Administration. These data are based on studies in which vandetanib has demonstrated clinical activity in a single-arm Phase II study in patients with metastatic hereditary MTC [19]. In this study, thirty patients (21 female; median age 50 years) received initial treatment with vandetanib 300 mg with a median duration of treatment of 172 days. Based on site investigator assessments, 20% (6/30) of patients experienced a partial response (duration of response 59–260 days) and another 30% (9/30) of patients experienced stable disease at 24 weeks, yielding a disease control rate of 50% (15/30). Similarly, in 19 patients, plasma calcitonin levels showed a >50% decrease from baseline that was maintained for at least 6 weeks. Based on these and other data, an international, randomized, placebo-controlled Phase III study of vandetanib monotherapy in metastatic MTC has been initiated and is currently ongoing.

In addition to vandetanib, several other novel RTK inhibitors are under investigation for treatment of RET-dependent thyroid carcinomas. Recently, Kim et al. have shown that sorafenib, a multikinase inhibitor of RTK, VEGFR, and BRAF kinase, inhibits proliferation of ATC cell lines and inhibits tumor angiogenesis via induction of endothelial apoptosis in an orthotopic anaplastic thyroid carcinoma xenograft model in nude mice [20]. Similarly, the orally administered multitarget tyrosine kinase inhibitor, sunitinib, has been shown to be a novel potent inhibitor of thyroid oncogenic RET/papillary thyroid cancer kinases [21]. Many of these agents which are earlier in the development pipeline are capable of inhibiting RET at subnanomolar concentrations and hold significant promise for the treatment and palliation of hereditary MTC [2].

### 3.2. EGFR as a Therapeutic Target

Epidermal growth factor (EGF) is a 6-kDa polypeptide which has been demonstrated to stimulate the proliferation of normal and malignant thyroid cells and inhibit cellular differentiation [22]. Overexpression of the EGF receptor (EGFR), a 170-kDa transmembrane glycoprotein tyrosine kinase, has been documented in various differentiated thyroid carcinomas [23, 24] and is thought to be essential in thyroid carcinoma proliferation and metastasis. Recently in a proteomics study of MTCs expressing *RET* germ-line mutations, Gorla and colleagues [25] demonstrated high-level expression of minimally phosphorylated EGFR in two separate MTC cell lines. These data, taken together with studies demonstrating that gefitinib and erlotinib, well known EGFR inhibitors, have resulted in objective tumor responses in patients with EGFR-overexpressing tumors, suggest that EGFR inhibitors might be beneficial for therapy of refractory or metastatic MTC. Interestingly, several of the novel RTK inhibitors—vandetanib, sorafenib, and sunitinib, for example—appear to be multitarget kinase inhibitors capable of nonspecific inhibition of multiple critical signaling pathways critical in MTC tumorigenesis. While vandetanib is currently in phase II clinical trials to measure efficacy and clinical response in patients with hereditary MTC, the question of whether the multitarget kinase inhibitors' lack of receptor specificity will be advantageous or disadvantageous remains unanswered.

### 3.3. Angiogenesis Inhibitors

Many proteins appear to be involved in the formation and maintenance of new blood vessels which support primary tumor growth and metastatic tumor deposits. One of these proteins, vascular endothelial growth factor (VEGF), stimulates angiogenesis by attaching to VEGF receptors on the endothelial cells, supporting new vessel stability [26]. Interestingly, several recent studies suggest that thyroid cancer cells demonstrate elevated levels of VEGF compared to normal controls.

To date, several phase II clinical trials have been completed in MTC for single agent small molecules which target the VEGF receptor. AG-013736 (Axitinib) is a potent small molecule inhibitor of VEGF receptors 1 through 3 which has been shown in a single arm multicenter trial to be associated with an overall partial response rate of 20% in patients with thyroid cancer of any histology that was resistant or not appropriate for ^131^I. Among patients with MTC, nearly 20% (2/11 patients) demonstrated radiographic partial responses while almost half (5/11 patients) of all MTC patients exhibited stabilization of disease. Therapy was well tolerated, with reported side-effects of fatigue, hypertension, and proteinuria [27]. A similar phase II trial of the multikinase inhibitor AMG-706 in MTC has been recently completed. In this study, patients with differentiated thyroid cancer or MTC received AMG-707 until disease progression or drug toxicity occurred. Although the results of the MTC cohort have not yet been reported, the results for the differentiated group have been presented and demonstrate partial tumor response by Recist criteria in 12% of patients while another 70% of patients demonstrated stable disease [28]. Clearly, these studies offer significant insight into the idea that targeting the soluble VEGF receptor may be a potential target for effective therapy in MTC.

## 4. Signal Transduction Proteins

### 4.1. Glycogen Synthase Kinase-3ß (GSK-3ß) in MTC

GSK-3ß is a multifunctional serine/threonine protein kinase that was first described as playing a role in the regulation of glycogen synthesis [29] and has since been shown to be an important regulator of cell proliferation and survival. In contrast to other kinases, GSK-3ß is highly active and nonphosphorylated in unstimulated cells; however, activity of the kinase is inhibited by phosphorylation of a single serine residue (Ser^9^) in response to signaling cascades, including the Raf-1/mitogen-regulated extracellular kinase (MEK)/extracellular regulated kinase (ERK) signaling pathway [29]. Recently, we have shown that Raf-1 activation in human MTC cells results in phosphorylation and subsequent inactivation of GSK-3ß [30]; likewise, inactivation of GSK-3ß by phosphorylation results in MTC growth inhibition both in vitro and in vivo. 

Several small molecule inhibitors of GSK-3ß are available and demonstrate promising results both in vitro and in vivo as potential targeted therapies for the treatment of MTC. We have recently shown that inactivation of GSK-3ß with lithium chloride (LiCl) and SB216763 results in MTC differentiation and cell growth inhibition; likewise, these small molecules have been shown to be associated with a significant decrease in NE markers such as human achaete-scute complex-like l (ASCL1) and chromogranin A (CgA) in cultured MTC cells [30]. In vivo studies in LiCl-treated MTC xenograft mice demonstrated a significant reduction in tumor volume compared with those treated with control [30]. LiCl has been utilized clinically for more than fifty years as an adjunctive psychiatric medication for the treatment of bipolar disorder and has shown only minimal adverse side effects. As such, the efficacy of LiCl therapy in patients with MTC is currently being investigated at our institution in phase II clinical trials.

### 4.2. The Notch1/Achaete-Scute Complex-Like 1 (ASCL1) Pathway

Notch1 is a multifunctional transmembrane receptor that regulates cellular differentiation, proliferation, and survival [31–33]. Binding of any one of the Notch ligands (e.g., DLL-1 or JAG-1) promotes a sequence of proteolytic cleavages resulting in the activated Notch intracellular domain (NICD). This activated form of Notch1 then translocates to the nucleus and binds with the DNA-binding protein complex CSL (C promoter-binding factor 1, suppressor of hairless and Lag-1), resulting in transactivation of various target genes like hairy enhancer of split-1 (HES-1).

 In human cancer cells, Notch1 acts as either a tumor suppressor or an oncogene. In many types of cancer, including pancreatic, colon, nonsmall cell lung cancer (NSCLC), cervical and renal cell cancer, Notch1 is upregulated; furthermore, it has been suggested that expression of Notch1 signaling prevents cellular differentiation and inhibits apoptosis in these cancer types. Conversely, Notch1 signaling is very minimal or absent in NE tumors such as small-cell lung cancer (SCLC), carcinoid cancer, and MTC [34]. In recent studies, we have shown that activation of doxycycline-inducible Notch1 in MTC cells significantly reduced MTC cellular growth and regulated calcitonin level in a dose-dependent fashion; furthermore, these changes were dependent on the amount of Notch1 protein [35]. These observations clearly suggest that Notch1 signaling proteins are conserved in MTC cells and support the idea that Notch1 activation may be a potential target to treat patients with MTC tumors. 

Until recently, methods for the delivery of activated Notch1 to tumor cells, aside from gene therapy, had been nonexistent. Recently, we have demonstrated that the histone deacetylase inhibitors valproic acid (VPA) and suberoyl *bis*-hydroxamic acid (SBHA) act as strong Notch1 activators in MTC cells [36, 37]. HDAC inhibitors represent a class of diverse molecules that modulate gene transcription by increasing histone acetylation; the resulting alteration in chromatin structure is believed to possess antineoplastic effects in preclinical and clinical studies in neuroblastoma cells and a variety of other cancers [38]. In our studies, SBHA and VPA treatment of MTC tumor cells resulted in dose-dependent induction of the Notch1-intracellular domain, the active form of the protein [36, 37]. Furthermore, with Notch1 activation there was a concomitant decrease in ASCL1 in vitro, a downstream target of Notch1 signaling. Currently, we are seeking to develop phase II clinical trials at our institution to determine the efficacy of these HDAC inhibitors as part of a comprehensive therapy in patients with MTC.

### 4.3. The Raf-1/Mitogen-Regulated Extracellular Kinase (MEK)/Extracellular-Regulated Kinase (ERK) Signaling Pathway

The Raf-1/MEK/ERK pathway has long been recognized for its role in tumor biology and specifically for its role in MTC tumor development [11]. Utilizing an estradiol-inducible estrogen receptor fused with the catalytic domain of the Raf-1 fusion protein, we have shown that activation of the Raf-1 pathway is associated with a reduction in NE hormones such as calcitonin and CgA [39]. More importantly, ectopic raf-1 expression led to significant MTC growth suppression both in vitro and in a mouse xenograft model of MTC [40–43]. More recently, Ning et al. [44] showed that the Raf-1 pathway regulates essential cell-cell contact molecules and the metastatic phenotype of MTC cells. These data clearly demonstrate that although activation of the Raf-1 signaling pathway is growth promoting in several cancers, in certain cell-specific subtypes, Raf-1 activation inhibits tumorigenesis. 

 Despite the fact that methods to deliver activated Raf-1 to MTC cells are limited, pharmacologic activators of the Raf-1 pathway are currently under investigation. ZM336372, originally identified as a potent and specific inhibitor of Raf isoforms [45], has been shown to paradoxically demonstrate a >100-fold induction of Raf-1 activity in NE cell culture systems [45, 46]. In MTC cells in vitro, Kunnimalaiyaan et al. [47] have demonstrated that ZM336372 treatment resulted in increasing Raf-1 activation as measured by phosphorylation of ERK1/2. Importantly, treatment with ZM336372 in the presence of small interfering RNA against Raf-1 resulted in an increase in Raf-1 production, suggesting that ZM336372 upregulates Raf-1 at the transcriptional level. While this is the first description of a novel compound capable of regulation the Raf-1 pathway in vitro, further studies into the in vivo effects of ZM336372 are ongoing.

## 5. Tumor Suppressor Genes and Nuclear Oncogenes

### 5.1. The p53 Tumor Suppressor Genes

It is believed that nearly 50% of all human malignancies are due to inactivating mutations of the p53 tumor suppressor gene; however, the significance of the p53 gene in MTC carcinogenesis is less clear [48]. In a cohort of nearly 100 patients, Saltman et al. clearly demonstrated the presence of aberrant p53 expression in more aggressive phenotypes of thyroid carcinoma; the study demonstrated a gradual increase in p53 immunopositivity rate along the spectrum of thyroid carcinoma progression with a statistically significant difference between well-differentiated and anaplastic phenotypes (0% versus 31.8%, resp.; *P* < .001) [49]. Similarly, using microdissection and genotyping, Sheikh et al. studied 11 cases of MTC for allelic losses in a panel of known tumor suppressor genes in an attempt to elucidate the molecular basis for clinical outcome. Among the tumor suppressor genes with the most frequent allelic losses, p53 demonstrated a 44% frequency of allelic loss. When combined with high-risk clinical variables, including advanced patient age and cervical lymph node status, this genotype accurately predicted nearly all patients in whom recurrence was likely [50]. Although frequency of allelic loss in tumor suppressor genes like p53 may provide a useful adjunctive prognostic test in MTC, there are currently no pharmacologic methods of selectively targeting this genotype.

### 5.2. The *c-myc*, *c-jun*, and *c-fos* Nuclear Proto-Oncogenes

The proto-oncogenes *c-myc*, *c-jun*, and *c-fos* regulate the production of nuclear transcription factors which activate the expression of target genes involved in the control of thyroid cell growth and differentiation [51]. Although no evidence for gene rearrangements or amplification of the nuclear proto-oncogenes has been noted in thyroid carcinomas, in human MTC samples, both a high frequency of *c-myc *allelic losses [50] and elevated levels of *c-myc* and *c-jun* mRNA have been demonstrated [52]. Correlation between the c-myc transcript levels and the thyroid carcinoma differentiation was reported, demonstrating that the less differentiated tumors are associated with a greater abundance of c-myc mRNA [53].

## 6. Conclusions

Although current treatment options for patients with metastatic and refractory MTC are limited, recent advances in molecular oncology have fostered the development of novel small molecules which target specific pathways that are thought to be essential in MTC tumorigenesis. Inhibitors of the activated *RET* proto-oncogene and other RTK inhibitors appear particularly promising, based on the high prevalence of mutated oncogenes and specific expression patterns in MTC [2]. Likewise, targeting angiogenesis in MTC with small-molecule VEGF inhibitors and multitarget kinase inhibitors is currently under investigation and has demonstrated promising results. Many of these newer RTK inhibitors are currently under investigation in a series of randomized trials. Furthermore, alterations in various cellular signaling pathways like the Notch1 and Raf-1/MEK/ERK pathways offer potential targets for novel small molecule regulators. As molecular techniques continue to be developed and the human genome sequenced, other target therapies will undoubtedly be developed and will enter the clinical setting, prompting patients with metastatic and refractory MTC to participate in clinical trials evaluating these novel therapies.

## Figures and Tables

**Table 1:** Targeted therapies currently in clinical trial development for MTC.

Drug	Target	Mechanism of action
Zactima (ZD6474)	VEGFR, RET, EGFR (HER1)	Tyrosine kinase inhibitor
XL184	VEGFR2, MET, RET	Tyrosine kinase inhibitor
Imatinib (STI571)	*bcr-abl*, PDGFR, C-KIT	Tyrosine kinase inhibitor
Sorafinib (BAY-43-9006)	BRAF, CRAF, VEGFR, RET, PDGFR	Tyrosine kinase inhibitor
Sunitinib (SU11248)	VEGFR, RET, PDGFR, C-KIT, CSF-1R, flt3	Tyrosine kinase inhibitor
AMG-706	VEGFR1-3, PDGFR, C-KIT	Tyrosine kinase inhibitor
Gefitinib	EGFR (HER1)	Tyrosine kinase inhibitor
Axitinib (AG-013736)	VEGFR, PDGFR, C-KIT	Tyrosine kinase inhibitor
Pazopanib (GW786034)	VEGFR1-3, PDGFR, C-KIT	Tyrosine kinase inhibitor
SAHA	Notch1, TNF-*α*, IL-1-*β*, IL-6, IFN-*γ*	HDAC inhibitor
Lithium	GSK-3ß	Unknown, suspected GSK3 inhibition, inositol depletion

## Abbreviations

VEGFR: Vascular endothelial growth factor receptor
RET: Rearranged during transfection
EGFR: Epidermal growth factor receptor
HER: Human epidermal growth factor receptor
MET: Hepatocyte growth factor receptor tyrosine kinase
C-KIT: Cluster of differentiation (CD) 117
BRAF: B-raf
CRAF: C-raf
PDGFR: Platelet-derived growth factor receptor
CSF-1R: Colony-stimulating receptor-1R
flt3: fms-like tyrosine kinase receptor-3
TNF: Tumor necrosis factor
IL: Interleukin
IFN: Interferon
GSK-3ß: Glycogen synthase kinase-3ß
HDAC: Histone deacetylase
SAHA: Suberoylanilide hydroxamic acid.
